# Emapalumab for severe cytokine release syndrome in solid tumor CAR-T: a case report

**DOI:** 10.3389/fonc.2025.1543622

**Published:** 2025-04-01

**Authors:** Tyler Ruemmele, Rodney Macedo, Mark N. Stein, Hei Ton Chan, Markus Y. Mapara, Céline F. Jacquemont, Ran Reshef

**Affiliations:** ^1^ Division of Hematology/Oncology, New-York Presbyterian Hospital/Columbia University Irving Medical Center, New York, NY, United States; ^2^ Herbert Irving Comprehensive Cancer Center, Columbia University Irving Medical Center, New York, NY, United States; ^3^ Columbia Center for Translational Immunology, Columbia University Irving Medical Center, New York, NY, United States; ^4^ Bellicum Pharmaceuticals, Inc., Houston, TX, United States

**Keywords:** chimeric antigen receptor (CAR T), cytokine release syndrome (CRS), emapalumab, interferon gamma (IFNγ), prostate stem cell antigen (PSCA), immune effector cell associated HLH-like syndrome (IEC-HS), prostate specific antigen (PSA), CAR (chimeric antigen receptor) T cells

## Abstract

Chimeric Antigen Receptor T (CAR-T) cell therapy significantly and rapidly changed the treatment paradigm for lymphoma, myeloma and leukemia, and the recent approvals of the first cellular immunotherapies in melanoma and synovial sarcoma demonstrate the potential success of this approach in solid tumors. Though the therapeutic potential of CAR-T is impressive, severe cytokine release syndrome (CRS) remains an ongoing challenge. Here we report a patient who received an investigational CAR-T product for metastatic castration-resistant prostate cancer who developed multi-drug refractory, life-threatening CRS, which was successfully treated with the interferon (IFN)-γ antagonist emapalumab. Within 12 hours after the first dose of emapalumab, there was a dramatic improvement in hemodynamic status and the patient was weaned off all four vasopressors. The hemodynamic improvement was associated with a decrease in IFN-γ and CXCL10 levels but no other cytokines. Not only was emapalumab the only drug effective at treating this case of refractory CRS, but it did not appear to reduce the activity of the CAR-T product, as the CAR-T vector copy numbers remained persistent and the patient’s PSA levels remained low. This case demonstrates the clinical use of emapalumab to treat refractory cytokine release syndrome in a solid tumor CAR-T while potentially preserving therapeutic efficacy of CAR-T therapy. Further studies with larger patient populations are needed to evaluate the use of emapalumab as a treatment for CRS.

## Introduction

Chimeric Antigen Receptor T (CAR-T) cell therapy is a form of genetically modified autologous or allogeneic immunotherapy armed with a receptor directed against an antigen on the surface of cancer cells. This novel treatment modality significantly and rapidly changed the treatment paradigm for lymphoma, myeloma and leukemia (1–4), and the recent approvals of the first cellular immunotherapies in melanoma and synovial sarcoma demonstrate the potential success of this approach in solid tumors (5, 6). Though the therapeutic potential of CAR-T is impressive, cytokine release syndrome (CRS) remains an ongoing challenge with rates as high as 92% in patients receiving CAR-T therapy (1). Treatment for CRS includes agents such as tocilizumab and anakinra (7–9), though some patients have severe CRS refractory to these interventions. Most research on the treatment of CRS in CAR-T therapy is based on CD19 and BCMA constructs targeting liquid tumors, while specific data on the treatment of CRS in solid tumors is largely lacking. This patient with prostate cancer received the investigational prostate stem cell antigen (PSCA) targeting autologous CAR-T product (BPX-601) on a Phase 1/2 clinical trial (NCT02744287). This particular CAR-T product was equipped with a cytoplasmic molecular “on switch” that allows re-activation of the CAR-T cells with weekly infusions of the dimerizing agent rimiducid starting on Day +7. This dimerization activates toll-like receptors and CD40 signaling pathways which triggers strong pro-survival, activation, and expansion signals. A separate report has been published on the results of this clinical trial (10).

Here, we report on a case of multi-drug refractory, life-threatening CRS, which was successfully treated with the interferon (IFN)-γ antagonist emapalumab.

## Case description

A 67-year-old patient was diagnosed with metastatic castration-resistant prostate cancer (mCRPC), T3bN0M1, Gleason score 8 with bone metastases involving S1 vertebra and left iliac bone in 2019. He was initially treated with degarelix, leuprolide, and apalutamide with a brief response. In 2020, olaparib was added and he received palliative radiation to metastatic lesions in the thoracic spine. In 2022, he received consolidative radiation therapy to the prostate and was treated with an ATR inhibitor on a clinical trial. He enrolled in the Phase 1/2 BPX-601 clinical trial using autologous prostate stem cell antigen (PSCA)-targeted CAR-T cells with weekly infusions of rimiducid (10). The patient’s T cells were collected, followed by bridging docetaxel as well as palliative radiation to the lumbar and sacral spine. Baseline prostate-specific antigen (PSA) level prior to clinical trial start date was 22.5 ng/mL.

He underwent lymphodepleting chemotherapy with fludarabine 30 mg/m^2^ and cyclophosphamide 500 mg/m^2^ on Day -5 through -3, followed by infusion of BPX-601 on Day 0. On Day +1, the patient developed fever consistent with Grade 1 cytokine release syndrome (CRS) as per ASTCT consensus grading, which progressed to Grade 2 (fever with hypoxia) on Day +2 and was treated with one dose of tocilizumab 8mg/kg. Blood and urine cultures were obtained, and he was started on broad spectrum antibiotics. On Day +3, he continued to have persistent fevers and hypoxia, as well as new fluid-responsive hypotension, still consistent with Grade 2 CRS. He was given a second and a third dose of tocilizumab, as well as dexamethasone 10mg x2. His CRP peaked at 96.77 mg/L, but ferritin was stable from baseline at 487 ng/mL. By Day +4, CRS was resolved. The patient had one Immune Effector Cell-Associated Encephalopathy (ICE) score of 9/10, which resolved without intervention and deemed not to be immune effector cell-associated neurotoxicity syndrome (ICANS), however levetiracetam was initiated and continued throughout the hospitalization.

On Day +5, the patient developed hematuria, dry eyes, and a diffuse papular rash with skin biopsy showing superficial perivascular mononuclear cell infiltrate consistent with a hypersensitivity reaction thought to be due to CAR-T “on-target/off-tumor” effect. The Day +7 rimiducid was delayed until symptoms improved.

The first dose of rimiducid was given on Day + 11. On Day +12, the patient developed a worsening rash, which was more pronounced on abdomen and trunk, and newly involving face and scalp. He also had worsening eye redness with blurry vision, as well as new swollen parotid and submandibular glands bilaterally.

On Day +13, he had recurrent fevers with severe hypotension (lowest BP 70/40) requiring a norepinephrine infusion, consistent with Grade 3 CRS, and was transferred to the ICU. He received fluid resuscitation, tocilizumab x 1 (fourth total dose), and dexamethasone 10mg every six hours. By Day +15, he was weaned off vasopressors and dexamethasone, and the skin rash, eye redness, and parotid gland swelling improved. He then was transferred out of the ICU.

**Figure 1:**
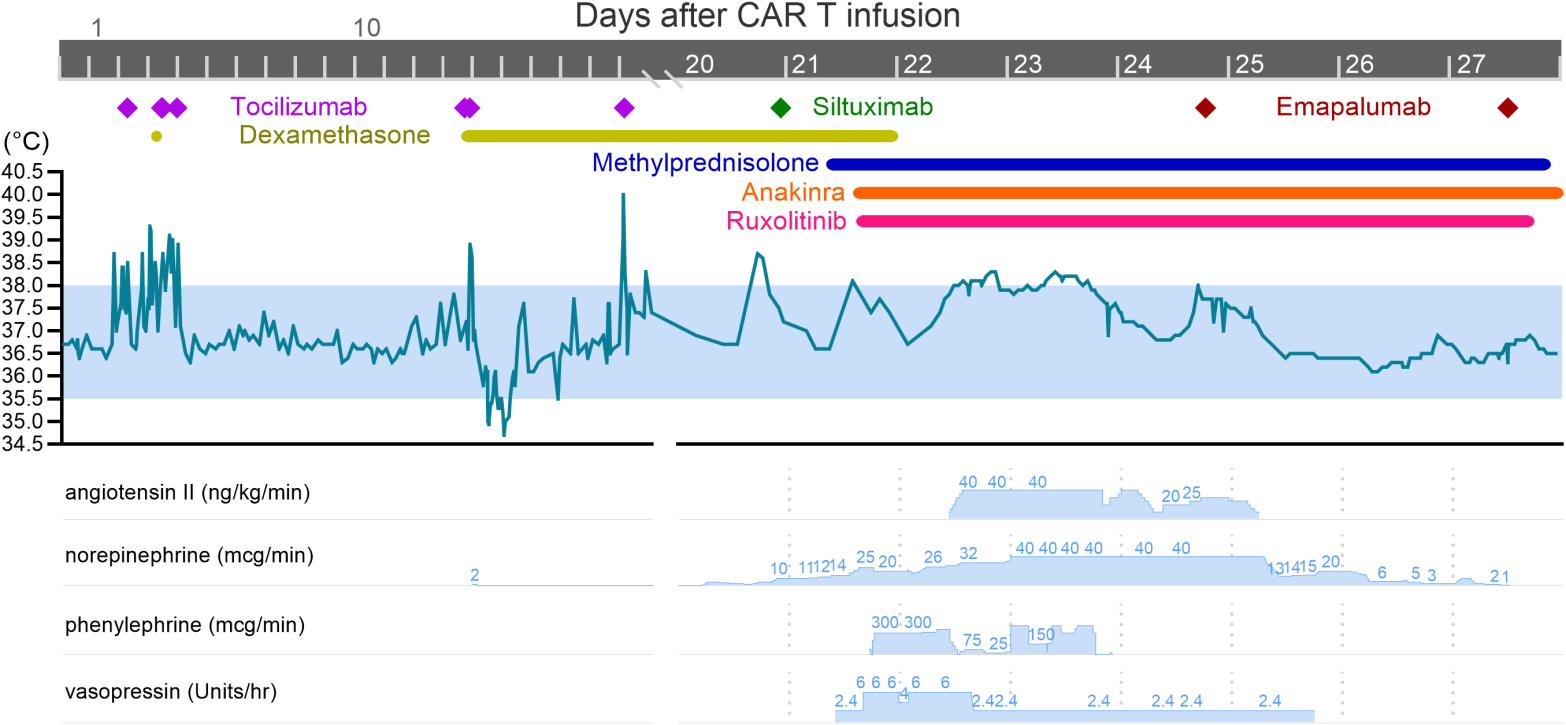
Effect of anti-cytokine therapy on fever and vasopressor requirement. The patient experienced three separate episodes of CRS: the first on Day +1, second on Day +13, and third and most severe on Day +19. After the second dose of rimiducid on Day +18, the patient rapidly decompensated and required multiple vasopressors. Note the lack of effect of siltuximab, methylprednisolone, anakinra, and ruxolitinib (all given on Day +21) on fever curve and vasopressor requirement. Emapalumab was first given on Day +24, which led to rapid decrease in temperature and vasopressor requirement.

Given rapid resolution of CRS and responsiveness to treatment, the second dose of weekly rimiducid was given on Day +18. The following day, the patient developed fevers, hypotension requiring a norepinephrine infusion, and hypoxia requiring 4L of oxygen via nasal cannula, consistent with Grade 3 CRS, and was transferred to the ICU. He was again given fluid boluses, tocilizumab (fifth total dose), and dexamethasone 10mg every 6hrs without improvement. Over the next two days, he developed a worsening vasopressor requirement (norepinephrine up to 40mcg/min, addition of vasopressin and phenylephrine) and required intubation for hypoxemic respiratory failure consistent with Grade 4 CRS. He had no evidence of ICANS and infectious workup was negative. He also had a rapid increase of ferritin to over 4,000 ng/mL and lactic acidosis (pH 7.12), however, his PSA level continued to decrease to 0.29. For rapidly progressive Grade 4 CRS without response to tocilizumab or dexamethasone, additional CRS treatment was initiated including siltuximab 11mg/kg once, methylprednisolone 1g twice daily, anakinra 200mg every 6 hours, and ruxolitinib 10mg twice daily on Day +21. The clinical course is summarized in **Figure 1**.

His hemodynamic status continued to deteriorate on Day +22. He developed multiorgan failure, disseminated intravascular coagulation (fibrinogen <50 mg/dL), and required continuous renal replacement therapy. He continued to have persistent fevers despite broad anti-cytokine therapy and high-dose steroids; norepinephrine, vasopressin, and phenylephrine were at or near their maximal doses; and angiotensin II was added. An abdominal ultrasound showed suspicion for acalculous cholecystitis, therefore an urgent percutaneous cholecystostomy tube was placed. Labs showed concern for immune effector cell-associated HLH-like syndrome (IEC-HS): he was pancyotopenic and transfusion dependent, ferritin peaked at 9,580 ng/mL, triglycerides elevated to 318 mg/dL, and soluble IL-2 receptor elevated to 93,326 pg/mL. A cytokine panel revealed that interferon (IFN)-γ was elevated to 1,786 pg/mL (baseline was <4.2 pg/mL on Day 0); thus, we decided to administer the IFN-γ antagonist emapalumab (Gamifant) 1mg/kg on Day +24. No other intervention was initiated.

**Figure 2:**
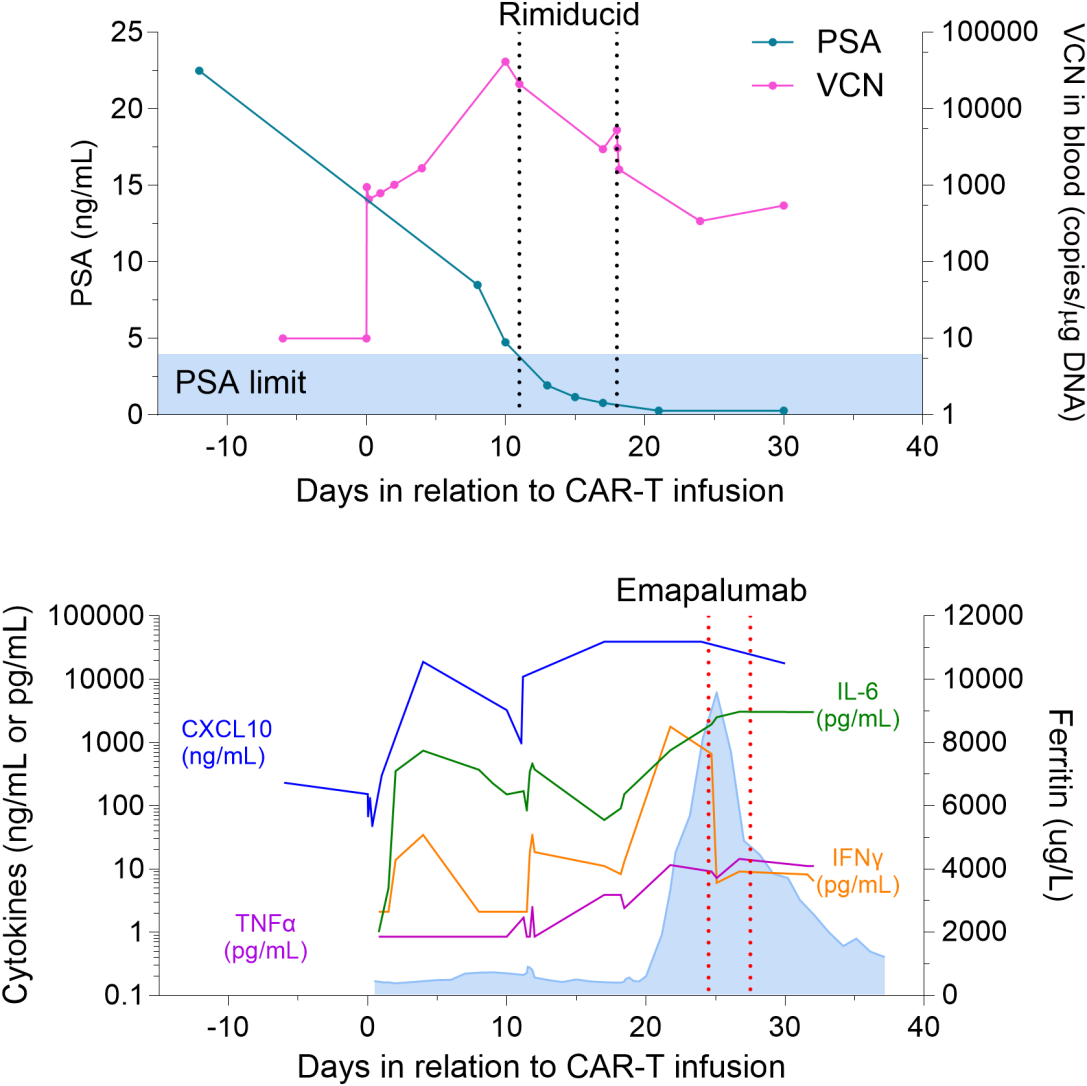
(Top panel) Prostate Specific Antigen (PSA) levels and CAR-T Vector Copy Numbers (VCN) in relation to date of cell infusion (Day 0) and rimiducid doses. Baseline PSA levels starting on Day -10 (before lymphodepleting chemotherapy) was 22.5 ng/mL and maintained at 0.29 ng/mL at time of death. CAR-T VCN remained persistent despite emapalumab administration on Day +24 and Day +27. (Bottom panel) Cytokine and ferritin levels in relation to date of cell infusion (Day 0) and emapalumab administration (Day +24 and Day +27). After the first dose of emapalumab, ferritin (blue shaded portion) decreased by over 50% from 9,580 ng/mL to 4,460 ng/mL, IFN-γ decreased by over 99% from 1,786.3 pg/mL to 6.0 pg/mL, and CXCL10 (IP-10), a downstream effector molecule in the IFN-γ signaling pathway, decreased 2-fold. IL-6 and TNF-α increased after emapalumab.

Within 12 hours of the first dose of emapalumab, he had a dramatic improvement in his hemodynamic status. Vasopressors were gradually withdrawn, he became afebrile, and oxygen requirement improved. Angiotensin and vasopressin were quickly weaned off, and he was maintained on low dose norepinephrine until Day +27. The second dose of emapalumab was given on Day +27, at which point he was normotensive, afebrile, and no longer on vasopressors (see **Figure 1**). Ferritin decreased by over 50% to 4,460 ng/mL, and IFN-γ decreased by over 99% from 1,786.3 pg/mL to 6.0 pg/mL (see **Figure 2**). CXCL10 (IP-10), a downstream effector molecule in the IFN-γ signaling pathway, decreased by 2-fold. Interestingly, other cytokines, such as IL-6 and TNF-α, either increased or did not significantly change. These trends were confirmed by separate lab studies conducted by the study sponsor (**Supplementary Figure S1**).

Despite hemodynamic and respiratory improvement, he had evidence of severe end-organ damage from prolonged shock manifested as anuric renal failure, digital ischemia and probable anoxic brain injury.

On Day +33, he developed bacteremia with vancomycin-resistant enterococcus, along with progressive encephalopathy and lactic acidosis. MRI brain showed multiple embolic strokes in frontal/parietal lobes. Given the significant multi-organ damage, his family decided to withdraw care and he passed away on D+37. He had no signs of active CRS at the time of death and his PSA dropped from baseline of 22.5 ng/mL before therapy to 0.29 ng/mL at time of death (see **Figure 2**).

## Discussion

Despite mortality, this case is remarkable for two reasons. First, emapalumab dramatically and rapidly led to complete resolution of CRS after failed attempts with tocilizumab, siltuximab, high dose steroids, anakinra, and ruxolitinib (see **Figure 1**). Levels of Inflammatory cytokines also increased despite administration of these medications (see **Figure 2**, **Supplementary Figure S1**). In contrast, emapalumab effectively reduced active IFN-γ levels and improved hemodynamic status. In particular, IFN-γ blockade seemed to clearly correlate with clinical improvement in fever curve and hemodynamic stability (i.e., reduction in vasopressor requirement). Other cytokines increased when CRS worsened, but did not decrease when CRS improved. This evidence supports the importance of IFN-γ as a primary driver of severe CRS and suggests that IFN-γ blockade can be used to effectively reverse CRS. To the best of our knowledge, ours is the first case report of emapalumab use in CAR-T for a solid tumor. Other studies have shown promise in IFN-γ blockade to treat severe CRS, though these are largely limited to the pediatric B-cell acute lymphoblastic leukemia population, with only one adult case report published to the best of our knowledge (11–13). These case reports also show mixed reduction in ferritin, IL-2, IL-6, IL-8, IL-10, TNF-α after administration of emapalumab, suggesting some differences in the overall inflammatory cascade in CRS induced by PSCA vs CD19-targeted CAR-T. IFN-γ blockade also has a key role in treating hemophagocytic lymphohistiocytosis (HLH), and HLH mouse models show that IFN-γ blockade has better survival outcomes compared to blockade of other cytokines (14–18). One study that showed the efficacy of emapalumab administration for pediatric primary HLH noted reduction in C-X-C motif chemokine ligand 9 (CXCL9), which is induced by IFN-γ (15). Our patient also had a decrease in a similar C-X-C motif chemokine ligand, CXCL10, after emapalumab administration (see **Figure 2**, **Supplementary Figure S1**).

Second, emapalumab did not appear to decrease activity of PSCA-targeting CAR-T therapy. Although follow up is short, the patient’s serum PSA dropped by nearly two orders of magnitude and was nearly undetectable (0.29 ng/mL) at the time of death. Most of the impressive PSA response was achieved prior to the first dose of emapalumab, however, our case is unique in that we were able to measure CAR-T vector copy numbers (VCN) in relation to multiple doses of emapalumab (see **Figure 2**). A persistence of CAR-T VCN in the peripheral blood, as well as the persistent decrease in PSA, suggest that emapalumab does not decrease efficacy of CAR-T cell therapy. However, it should be noted that the pre-emapalumab PSA level and post-emapalumab PSA level was the same (0.29 ng/mL). Decrease in PSA was similar to other patients in this trial, with 44.4% of mCRPC patients achieving a reduction of 90% or greater (10). Preclinical studies demonstrate the effective use of IFN-γ blockade while preserving CAR-T cytotoxicity in hematologic malignancies (19, 20), though a critical role of the IFN-γ signaling pathway in mediating CAR-T cytotoxicity in solid tumors has been demonstrated both *in vitro* and in immunocompromised mice (21). This suggests a key difference between hematologic and solid tumor CAR-T therapies, although the role of IFN-γ in CAR-T in humans needs further study and the specific clinical scenario presented in this case report (i.e., PSCA target, use of an activation switch, prostate cancer microenvironment) may limit a broad conclusion.

It is thought that CRS typically occurs at the same time as CAR-T cell expansion due to an increase in cytokines levels (22). In this particular trial, the dimerizing agent rimiducid was administered to assist with CAR-T expansion. Rimiducid was given on Day +11 and Day +18, which increased CAR-T vector copy numbers to 21,033 and 5,299 copies/µg, respectively (see **Figure 2**), but also ultimately led to Grade 3 and Grade 4 CRS, respectively. Cytokine levels increased after each administration of rimiducid (see **Figure 2**, **Supplementary Figure S1**), which correlated with worsening CRS. Of note, all patients with mCRPC in this trial had an increase in pro-inflammatory cytokines and clinical evidence of CRS after the first dose of rimiducid (10). The contribution of “off-tumor” antigen burden to the severity of CRS is possible in this case, given the expression of PSCA in certain healthy tissues and the off-target manifestation of a disseminated skin rash in this patient. This case highlights the critical need for better predictors of severe toxicity that can be used in real time to guide patient management.

Interestingly, emapalumab had a dramatic effect after failure of ruxolitinib. While both agents act on the IFN-γ-JAK-STAT pathway, there are key differences. First, ruxolitinib is only available orally and its absorption in the setting of profound circulatory shock may be inadequate. Second, ruxolitinib has an effect on signaling of multiple cytokines which may be suboptimal if the pathogenesis is primarily driven by IFN-γ. Lastly, IFN-γ triggers non-canonical STAT-independent pathways that affect cellular metabolism, histone modification and NF-kB signaling, which may explain a selective response to emapalumab (23).

A limitation of our study is that this is a case report with a PSCA targeting CAR-T product under clinical investigation. Further studies with larger patient populations should be conducted before extrapolating these findings to other types of solid tumor and hematologic malignancy CAR-T products. In this case, CRS appeared to dramatically and rapidly improve after emapalumab administration, but methylprednisolone, anakinra, and ruxolitinib all were continued during this resolution. The possibility of synergistic or cumulative effects from these other agents cannot be entirely ruled out. Despite resolution of CRS and sustained PSA reduction, the patient unfortunately did not survive and the duration of response was not evaluated past Day +30. With multiple immunosuppressive medications given to treat severe CRS, this patient developed a severe bacteremia, which highlights the need for further studies to evaluate effect of CRS treatment on infection risk and overall survival.

In conclusion, emapalumab appears to be an effective agent in treating severe CRS while preserving CAR-T persistence. Further research is needed to help understand the potential of IFN-γ blockade in CRS and its impact on anti-tumor efficacy.

## Data Availability

The original contributions presented in the study are included in the article/**Supplementary Material**. Further inquiries can be directed to the corresponding authors.

## Supplementary material

Supplementary Figure 1 Cytokine and ferritin levels (Source: Bellicum Inc.) in relation to date of cell infusion (Day 0) and emapalumab administration. IFN-γ CXCL10, CM-CSF decreased after emapalumab, while most other cytokines either increased or did not significantly change.
