# Supplementary figures and images for: Emapalumab for severe cytokine release syndrome in solid tumor CAR-T: a case report
Source: Front Oncol. 2025 Apr 1;15:1543622. doi: 10.3389/fonc.2025.1543622 (PMC11997384; doi:10.3389/fonc.2025.1543622)

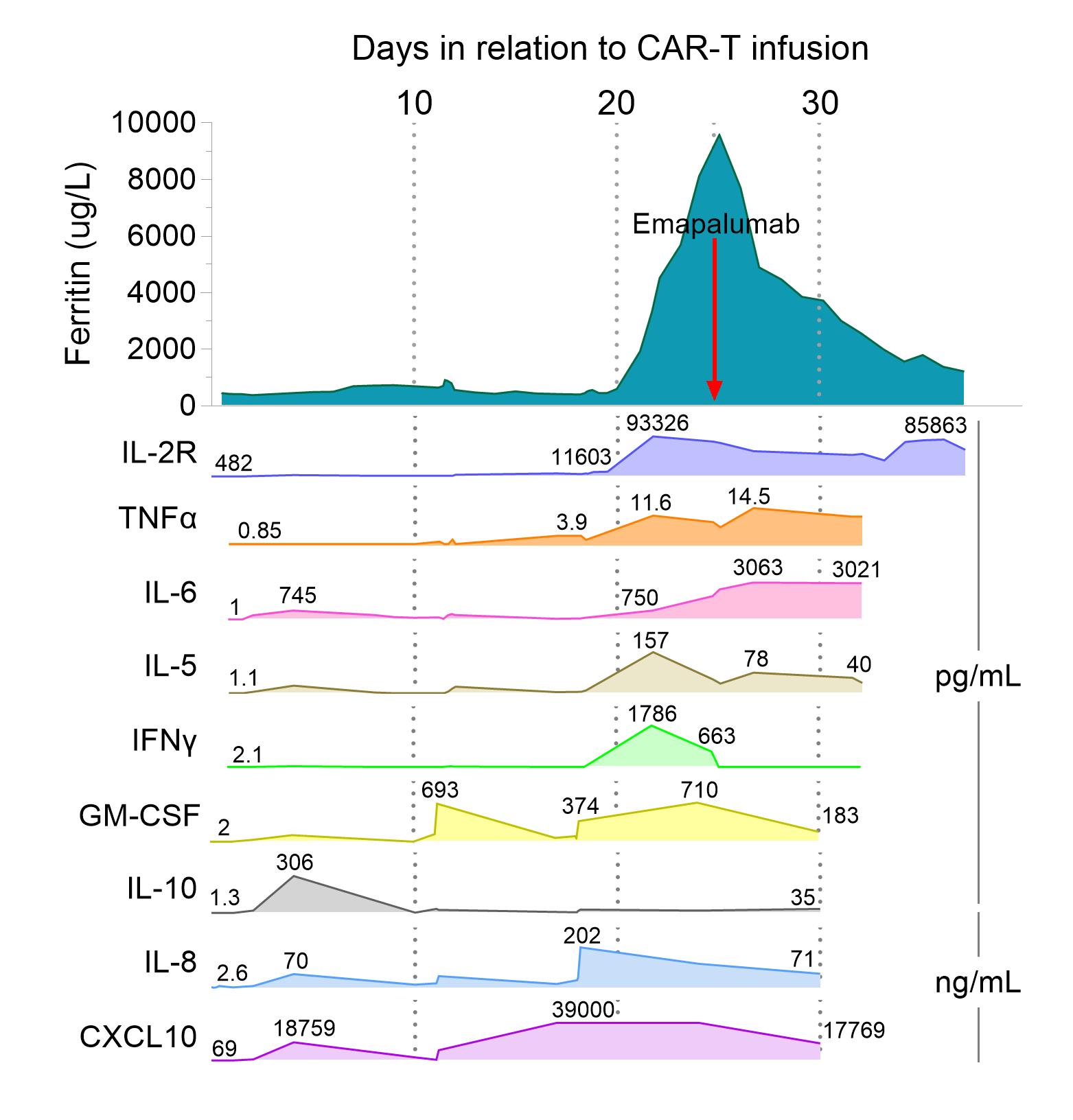

Supplement: Supplementary Figure 1 — Cytokine and ferritin levels (Source: Bellicum Inc.) in relation to date of cell infusion (Day 0) and emapalumab administration. IFN-γ CXCL10, CM-CSF decreased after emapalumab, while most other cytokines either increased or did not significantly change. [file Image1.tif]
